# (2*R*,3*R*)-*N*-(4-Chloro­phen­yl)-2,3-dihydr­oxy-*N*′-(5-phenyl-1,3,4-thia­diazol-2-yl)succinamide

**DOI:** 10.1107/S1600536810007919

**Published:** 2010-03-06

**Authors:** Hui-Ming Huang, Gen-Lin Chen, Min Li, Guo-Gang Tu, Cheng-Mei Liu

**Affiliations:** a State Key Laboratory of Food Science and Technology, Nanchang University, 330047 Nanchang, Jiangxi, People’s Republic of China; b Nanchang University School of Pharmaceutical Science, 330006 Nanchang, Jiangxi, People’s Republic of China

## Abstract

In the structure of the title compound, C_18_H_15_ClN_4_O_4_S, the dihedral angle between the two benzene rings is 1.4 (3)°. The angle between the phenyl ring and thia­diazole ring is 5.8 (4)°. The conformations of the N—H and C=O bonds are *anti* with respect to each other. In the crystal structure, mol­ecules are linked by inter­molecular O—H⋯N, N—H⋯O and O—H⋯O hydrogen bonds, forming a three-dimensional network.

## Related literature

For the synthesis, see: Marson & Melling (2005 ▶); Tu *et al.* (2008 ▶); Shriner & Furrow (1955 ▶). For related structures, see: Watadani *et al.* (2005 ▶); Li *et al.* (2008 ▶).
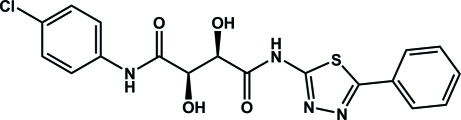


## Experimental

### 

#### Crystal data

C_18_H_15_ClN_4_O_4_S
*M* *_r_* = 418.85
Monoclinic, 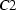

*a* = 41.381 (3) Å
*b* = 5.1744 (5) Å
*c* = 8.7442 (9) Å
β = 98.315 (1)°
*V* = 1852.6 (3) Å^3^
*Z* = 4
Mo *K*α radiation
μ = 0.35 mm^−1^
*T* = 298 K
0.46 × 0.40 × 0.11 mm

#### Data collection

Bruker SMART CCD area-detector diffractometer
Absorption correction: multi-scan (*SADABS*; Sheldrick, 1996 ▶) *T*_min_ = 0.855, *T*_max_ = 0.962
4632 measured reflections
2697 independent reflections
2319 reflections with *I* > 2σ(*I*)
*R*_int_ = 0.022

#### Refinement

*R*[*F* ^2^ > 2σ(*F* ^2^)] = 0.036
*wR*(*F* ^2^) = 0.091
*S* = 1.04
2697 reflections
253 parameters
1 restraint
H-atom parameters constrained
Δρ_max_ = 0.19 e Å^−3^
Δρ_min_ = −0.15 e Å^−3^
Absolute structure: Flack (1983 ▶), 863 Friedel pairs
Flack parameter: 0.03 (8)

### 

Data collection: *SMART* (Bruker, 2001 ▶); cell refinement: *SAINT* (Bruker, 2001 ▶); data reduction: *SAINT*; program(s) used to solve structure: *SHELXS97* (Sheldrick, 2008 ▶); program(s) used to refine structure: *SHELXL97* (Sheldrick, 2008 ▶); molecular graphics: *SHELXTL* (Sheldrick, 2008 ▶); software used to prepare material for publication: *SHELXTL*.

## Supplementary Material

Crystal structure: contains datablocks I, global. DOI: 10.1107/S1600536810007919/im2182sup1.cif

Structure factors: contains datablocks I. DOI: 10.1107/S1600536810007919/im2182Isup2.hkl

Additional supplementary materials:  crystallographic information; 3D view; checkCIF report

## Figures and Tables

**Table 1:** Hydrogen-bond geometry (Å, °)

*D*—H⋯*A*	*D*—H	H⋯*A*	*D*⋯*A*	*D*—H⋯*A*
O3—H3⋯N1^i^	0.82	1.95	2.733 (3)	159
N3—H3*B*⋯O3^ii^	0.86	2.35	3.061 (4)	140
O4—H4⋯O1^iii^	0.82	2.02	2.790 (3)	155
N4—H4*A*⋯O2^iv^	0.86	2.17	2.951 (4)	151

Symmetry codes: (i) 
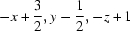
; (ii) 
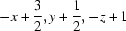
; (iii) 
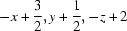
; (iv) 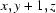.

**Fig. 1.**
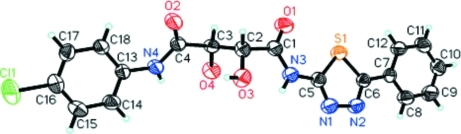
Molecular structure of the title compound. Displacement ellipsoids are drawn at the 50% probability level.

**Fig. 2.**
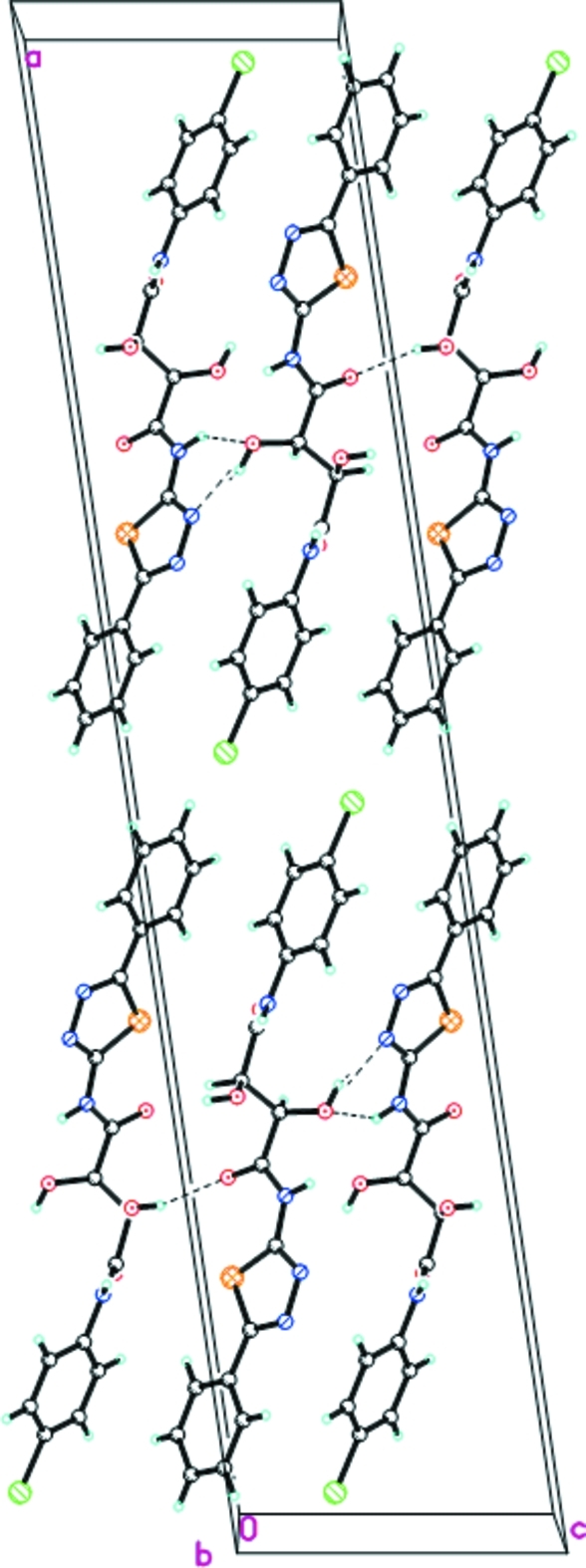
The crystal packing of title compound, viewed along the b axis with hydrogen bonds drawn as dashed lines.

## supplementary crystallographic information

### Comment

The present tartaric acid derivate is in continuation to our previously reported crystal structure of thiadiazole scaffold compounds (Li *et al.*, 2008). The title compound (Fig. 1) was synthesized according to literature procedures (Marson & Melling 2005; Tu *et al.*, 2008; Shriner & Furrow 1995) and crystallized in the monoclinic crystal system. The dihedral angle between the two benzene rings is 1.4 (3)°; the angle between the benzene ring (C7-C12) and thiadiazole ring is 5.8 (4)°. The conformations of the N—H and C=O bonds are *anti* with respect to each other. Bond lengths and angles are in normal ranges and comparable to those in related structures (Watadani *et al.*, 2005). In the crystal structure, molecules are linked by intermolecular O—H···N, N—H···O and O—H···O hydrogen bonds forming a three-dimensional network (Table 1, Figure 2).

### Experimental

To a solution of 2-amino-5-phenyl-1,3,4-thiadiazole (10 mmol) in THF was added diacetyl-*L*-tartaric anhydride (12 mmol). After the mixture was stirred at room temperature for 16 h, *N,N*-dicyclohexylcarbodiimide (9 mmol) and *p*-chloroaniline (9 mmol) in THF were added to the mixture. The reaction mixture was stirred at room temperature overnight. After insoluble material was filtered off the filtrate was evaporated in *vacuo*. The residual was hydrolyzed by a solution of K_2_CO_3_ in methanol at 65°C for 2 h and recrystallized from THF to afford the target compound. Yield: 3.06 g, 81%, m.p. 221-222°C. Colorless block-shaped single crystals of the title compound suitable for X-ray diffraction analysis precipitated after several days.

### Refinement

H atoms were positioned geometrically and refined using a riding model, with C—H = 0.93–0.96 Å, O—H = 0.82–0.85 Å and N—H = 0.86 Å, *U*_iso_(H) = 1.2*U*_eq_(C,N), and 1.5*U*_eq_(O). The absolute configuration is undoubtly as described since enantiomerically pure starting compounds were used and the reaction conditions are not considered to lead to racemisation or inversion.

### Crystal data

**Table:**

C_18_H_15_ClN_4_O_4_S	*F*(000) = 864
*M**_r_* = 418.85	*D*_x_ = 1.502 Mg m^−^^3^
Monoclinic, *C*2	Mo *K*α radiation, λ = 0.71073 Å
Hall symbol: C 2y	Cell parameters from 2277 reflections
*a* = 41.381 (3) Å	θ = 2.4–27.9°
*b* = 5.1744 (5) Å	µ = 0.35 mm^−^^1^
*c* = 8.7442 (9) Å	*T* = 298 K
β = 98.315 (1)°	Block, colourless
*V* = 1852.6 (3) Å^3^	0.46 × 0.40 × 0.11 mm
*Z* = 4	

### Data collection

**Table:**

Bruker SMART CCD area-detector diffractometer	2697 independent reflections
Radiation source: fine-focus sealed tube	2319 reflections with *I* > 2σ(*I*)
graphite	*R*_int_ = 0.022
phi and ω scans	θ_max_ = 25.0°, θ_min_ = 2.0°
Absorption correction: multi-scan (*SADABS*; Sheldrick, 1996)	*h* = −44→48
*T*_min_ = 0.855, *T*_max_ = 0.962	*k* = −6→4
4632 measured reflections	*l* = −10→10

### Refinement

**Table:**

Refinement on *F*^2^	Secondary atom site location: difference Fourier map
Least-squares matrix: full	Hydrogen site location: inferred from neighbouring sites
*R*[*F*^2^ > 2σ(*F*^2^)] = 0.036	H-atom parameters constrained
*wR*(*F*^2^) = 0.091	*w* = 1/[σ^2^(*F*_o_^2^) + (0.0471*P*)^2^ + 0.5765*P*] where *P* = (*F*_o_^2^ + 2*F*_c_^2^)/3
*S* = 1.04	(Δ/σ)_max_ = 0.001
2697 reflections	Δρ_max_ = 0.19 e Å^−^^3^
253 parameters	Δρ_min_ = −0.15 e Å^−^^3^
1 restraint	Absolute structure: Flack (1983), 863 Friedel pairs
Primary atom site location: structure-invariant direct methods	Flack parameter: 0.03 (8)

### Special details

**Table:**

Geometry. All esds (except the esd in the dihedral angle between two l.s. planes) are estimated using the full covariance matrix. The cell esds are taken into account individually in the estimation of esds in distances, angles and torsion angles; correlations between esds in cell parameters are only used when they are defined by crystal symmetry. An approximate (isotropic) treatment of cell esds is used for estimating esds involving l.s. planes.
Refinement. Refinement of *F*^2^ against ALL reflections. The weighted *R*-factor wR and goodness of fit *S* are based on *F*^2^, conventional *R*-factors *R* are based on *F*, with *F* set to zero for negative *F*^2^. The threshold expression of *F*^2^ > σ(*F*^2^) is used only for calculating *R*-factors(gt) etc. and is not relevant to the choice of reflections for refinement. *R*-factors based on *F*^2^ are statistically about twice as large as those based on *F*, and *R*- factors based on ALL data will be even larger.

### Fractional atomic coordinates and isotropic or equivalent isotropic displacement parameters (Å^2^)

**Table:**

	*x*	*y*	*z*	*U*_iso_*/*U*_eq_	
Cl1	0.51651 (2)	0.5674 (3)	0.31490 (11)	0.0776 (4)	
N1	0.82422 (6)	0.6761 (7)	0.6878 (3)	0.0533 (8)	
N2	0.85606 (6)	0.7102 (6)	0.7553 (3)	0.0510 (8)	
N3	0.77780 (5)	0.4311 (5)	0.7013 (3)	0.0438 (7)	
H3B	0.7686	0.5112	0.6203	0.053*	
N4	0.64966 (5)	0.5279 (6)	0.6689 (3)	0.0412 (6)	
H4A	0.6589	0.6749	0.6906	0.049*	
O1	0.76958 (5)	0.1304 (5)	0.8778 (3)	0.0567 (7)	
O2	0.65669 (5)	0.0938 (5)	0.7001 (3)	0.0587 (6)	
O3	0.72236 (4)	0.3604 (6)	0.5396 (2)	0.0508 (6)	
H3	0.7052	0.3206	0.4860	0.076*	
O4	0.70977 (5)	0.6252 (4)	0.8040 (2)	0.0485 (6)	
H4	0.7120	0.6645	0.8958	0.073*	
S1	0.833503 (16)	0.34609 (19)	0.90607 (8)	0.0471 (2)	
C1	0.75941 (7)	0.2576 (6)	0.7638 (3)	0.0394 (8)	
C2	0.72451 (6)	0.2352 (7)	0.6839 (3)	0.0404 (8)	
H2	0.7187	0.0524	0.6681	0.048*	
C3	0.70219 (6)	0.3600 (7)	0.7875 (3)	0.0378 (6)	
H3A	0.7059	0.2776	0.8894	0.045*	
C4	0.66679 (6)	0.3166 (7)	0.7157 (3)	0.0386 (7)	
C5	0.80992 (7)	0.4943 (7)	0.7539 (3)	0.0412 (8)	
C6	0.86447 (6)	0.5530 (7)	0.8697 (3)	0.0374 (7)	
C7	0.89748 (6)	0.5497 (7)	0.9587 (3)	0.0363 (7)	
C8	0.92006 (7)	0.7319 (7)	0.9290 (4)	0.0510 (9)	
H8	0.9142	0.8595	0.8553	0.061*	
C9	0.95150 (8)	0.7257 (8)	1.0084 (4)	0.0590 (10)	
H9	0.9667	0.8478	0.9869	0.071*	
C10	0.96029 (7)	0.5415 (8)	1.1180 (4)	0.0557 (9)	
H10	0.9814	0.5391	1.1716	0.067*	
C11	0.93800 (7)	0.3592 (9)	1.1492 (4)	0.0558 (9)	
H11	0.9441	0.2335	1.2238	0.067*	
C12	0.90653 (7)	0.3624 (8)	1.0698 (3)	0.0469 (8)	
H12	0.8915	0.2389	1.0911	0.056*	
C13	0.61733 (6)	0.5301 (7)	0.5857 (3)	0.0373 (7)	
C14	0.60929 (7)	0.7228 (7)	0.4779 (3)	0.0464 (8)	
H14	0.6250	0.8437	0.4602	0.056*	
C15	0.57817 (8)	0.7379 (7)	0.3959 (4)	0.0505 (9)	
H15	0.5727	0.8704	0.3251	0.061*	
C16	0.55547 (7)	0.5544 (7)	0.4205 (3)	0.0465 (8)	
C17	0.56310 (6)	0.3612 (8)	0.5266 (4)	0.0505 (8)	
H17	0.5475	0.2388	0.5428	0.061*	
C18	0.59436 (6)	0.3490 (8)	0.6101 (3)	0.0455 (7)	
H18	0.5997	0.2183	0.6824	0.055*	

### Atomic displacement parameters (Å^2^)

**Table:**

	*U*^11^	*U*^22^	*U*^33^	*U*^12^	*U*^13^	*U*^23^
Cl1	0.0454 (5)	0.1052 (10)	0.0745 (6)	0.0098 (6)	−0.0174 (4)	−0.0027 (6)
N1	0.0330 (13)	0.072 (2)	0.0507 (15)	−0.0065 (14)	−0.0070 (11)	0.0187 (15)
N2	0.0342 (13)	0.067 (2)	0.0487 (15)	−0.0096 (14)	−0.0045 (11)	0.0138 (15)
N3	0.0319 (12)	0.057 (2)	0.0389 (13)	−0.0026 (12)	−0.0077 (10)	0.0075 (12)
N4	0.0353 (12)	0.0280 (14)	0.0570 (14)	−0.0032 (12)	−0.0039 (10)	−0.0028 (13)
O1	0.0445 (11)	0.0624 (18)	0.0572 (13)	−0.0051 (12)	−0.0125 (10)	0.0186 (13)
O2	0.0386 (11)	0.0314 (14)	0.1006 (18)	−0.0036 (11)	−0.0086 (11)	0.0009 (13)
O3	0.0376 (10)	0.0773 (17)	0.0334 (10)	−0.0068 (13)	−0.0091 (8)	−0.0006 (12)
O4	0.0474 (12)	0.0397 (14)	0.0545 (12)	−0.0058 (11)	−0.0056 (9)	−0.0140 (10)
S1	0.0327 (3)	0.0557 (6)	0.0486 (4)	−0.0064 (4)	−0.0085 (3)	0.0129 (4)
C1	0.0365 (15)	0.043 (2)	0.0369 (15)	0.0019 (14)	−0.0022 (13)	−0.0028 (14)
C2	0.0332 (15)	0.042 (2)	0.0422 (16)	−0.0023 (14)	−0.0063 (12)	−0.0065 (15)
C3	0.0371 (14)	0.0351 (17)	0.0385 (14)	−0.0033 (16)	−0.0036 (11)	0.0013 (16)
C4	0.0350 (14)	0.0327 (19)	0.0469 (15)	−0.0010 (15)	0.0023 (12)	−0.0036 (15)
C5	0.0333 (14)	0.053 (2)	0.0356 (14)	0.0019 (15)	−0.0020 (12)	0.0008 (15)
C6	0.0333 (14)	0.043 (2)	0.0347 (14)	0.0013 (15)	0.0020 (11)	−0.0008 (15)
C7	0.0284 (13)	0.042 (2)	0.0374 (14)	−0.0007 (14)	0.0016 (11)	−0.0050 (15)
C8	0.0420 (17)	0.055 (2)	0.0530 (19)	−0.0029 (17)	−0.0049 (14)	0.0103 (17)
C9	0.0375 (17)	0.066 (3)	0.071 (2)	−0.0122 (17)	−0.0017 (16)	0.007 (2)
C10	0.0316 (15)	0.062 (3)	0.068 (2)	0.0018 (18)	−0.0092 (14)	0.000 (2)
C11	0.0433 (16)	0.061 (2)	0.0585 (19)	0.007 (2)	−0.0093 (14)	0.012 (2)
C12	0.0386 (14)	0.048 (2)	0.0519 (17)	−0.0052 (18)	−0.0012 (13)	0.0067 (19)
C13	0.0312 (13)	0.0354 (18)	0.0439 (15)	0.0022 (14)	0.0011 (11)	−0.0047 (15)
C14	0.0459 (17)	0.040 (2)	0.0516 (18)	−0.0033 (15)	0.0001 (14)	0.0037 (16)
C15	0.0555 (19)	0.046 (2)	0.0463 (17)	0.0077 (17)	−0.0040 (15)	0.0056 (16)
C16	0.0377 (15)	0.053 (2)	0.0461 (16)	0.0080 (18)	−0.0027 (13)	−0.0106 (17)
C17	0.0324 (14)	0.047 (2)	0.071 (2)	−0.0044 (18)	0.0031 (14)	−0.001 (2)
C18	0.0354 (14)	0.0396 (19)	0.0599 (18)	0.0019 (17)	0.0014 (13)	0.0069 (19)

### Geometric parameters (Å, °)

**Table:**

Cl1—C16	1.739 (3)	C6—C7	1.471 (3)
N1—C5	1.292 (4)	C7—C8	1.379 (4)
N1—N2	1.375 (3)	C7—C12	1.385 (5)
N2—C6	1.297 (4)	C8—C9	1.384 (4)
N3—C1	1.343 (4)	C8—H8	0.9300
N3—C5	1.381 (3)	C9—C10	1.363 (5)
N3—H3B	0.8600	C9—H9	0.9300
N4—C4	1.335 (4)	C10—C11	1.374 (5)
N4—C13	1.427 (3)	C10—H10	0.9300
N4—H4A	0.8600	C11—C12	1.384 (4)
O1—C1	1.218 (3)	C11—H11	0.9300
O2—C4	1.227 (4)	C12—H12	0.9300
O3—C2	1.410 (4)	C13—C18	1.373 (4)
O3—H3	0.8200	C13—C14	1.380 (5)
O4—C3	1.411 (4)	C14—C15	1.383 (4)
O4—H4	0.8200	C14—H14	0.9300
S1—C5	1.713 (3)	C15—C16	1.374 (5)
S1—C6	1.734 (3)	C15—H15	0.9300
C1—C2	1.515 (4)	C16—C17	1.369 (5)
C2—C3	1.528 (4)	C17—C18	1.392 (4)
C2—H2	0.9800	C17—H17	0.9300
C3—C4	1.525 (3)	C18—H18	0.9300
C3—H3A	0.9800		
			
C5—N1—N2	112.0 (2)	C8—C7—C6	119.7 (3)
C6—N2—N1	112.6 (3)	C12—C7—C6	121.0 (3)
C1—N3—C5	126.7 (2)	C7—C8—C9	120.2 (3)
C1—N3—H3B	116.7	C7—C8—H8	119.9
C5—N3—H3B	116.7	C9—C8—H8	119.9
C4—N4—C13	125.5 (3)	C10—C9—C8	120.3 (3)
C4—N4—H4A	117.3	C10—C9—H9	119.8
C13—N4—H4A	117.3	C8—C9—H9	119.8
C2—O3—H3	109.5	C9—C10—C11	120.1 (3)
C3—O4—H4	109.5	C9—C10—H10	120.0
C5—S1—C6	86.24 (14)	C11—C10—H10	120.0
O1—C1—N3	123.0 (3)	C10—C11—C12	120.1 (3)
O1—C1—C2	122.0 (3)	C10—C11—H11	119.9
N3—C1—C2	115.0 (2)	C12—C11—H11	119.9
O3—C2—C1	108.1 (2)	C11—C12—C7	120.0 (3)
O3—C2—C3	111.8 (3)	C11—C12—H12	120.0
C1—C2—C3	108.2 (2)	C7—C12—H12	120.0
O3—C2—H2	109.6	C18—C13—C14	119.7 (3)
C1—C2—H2	109.6	C18—C13—N4	122.3 (3)
C3—C2—H2	109.6	C14—C13—N4	118.0 (3)
O4—C3—C4	111.8 (3)	C13—C14—C15	120.6 (3)
O4—C3—C2	109.1 (2)	C13—C14—H14	119.7
C4—C3—C2	108.7 (2)	C15—C14—H14	119.7
O4—C3—H3A	109.0	C16—C15—C14	119.1 (3)
C4—C3—H3A	109.0	C16—C15—H15	120.4
C2—C3—H3A	109.0	C14—C15—H15	120.4
O2—C4—N4	125.3 (3)	C17—C16—C15	121.0 (3)
O2—C4—C3	118.4 (3)	C17—C16—Cl1	119.6 (3)
N4—C4—C3	116.2 (3)	C15—C16—Cl1	119.5 (3)
N1—C5—N3	120.3 (3)	C16—C17—C18	119.6 (3)
N1—C5—S1	115.3 (2)	C16—C17—H17	120.2
N3—C5—S1	124.4 (3)	C18—C17—H17	120.2
N2—C6—C7	122.7 (3)	C13—C18—C17	120.0 (3)
N2—C6—S1	113.92 (19)	C13—C18—H18	120.0
C7—C6—S1	123.4 (2)	C17—C18—H18	120.0
C8—C7—C12	119.3 (2)		
			
C5—N1—N2—C6	−0.1 (4)	C5—S1—C6—C7	−179.0 (3)
C5—N3—C1—O1	−0.5 (5)	N2—C6—C7—C8	5.0 (5)
C5—N3—C1—C2	178.1 (3)	S1—C6—C7—C8	−175.8 (2)
O1—C1—C2—O3	−167.3 (3)	N2—C6—C7—C12	−173.5 (3)
N3—C1—C2—O3	14.1 (4)	S1—C6—C7—C12	5.8 (4)
O1—C1—C2—C3	71.5 (4)	C12—C7—C8—C9	0.6 (5)
N3—C1—C2—C3	−107.1 (3)	C6—C7—C8—C9	−177.9 (3)
O3—C2—C3—O4	−55.8 (3)	C7—C8—C9—C10	−0.8 (6)
C1—C2—C3—O4	63.0 (3)	C8—C9—C10—C11	0.6 (6)
O3—C2—C3—C4	66.4 (3)	C9—C10—C11—C12	−0.2 (6)
C1—C2—C3—C4	−174.8 (3)	C10—C11—C12—C7	0.0 (6)
C13—N4—C4—O2	−3.5 (5)	C8—C7—C12—C11	−0.2 (5)
C13—N4—C4—C3	173.6 (2)	C6—C7—C12—C11	178.3 (3)
O4—C3—C4—O2	−177.4 (3)	C4—N4—C13—C18	34.5 (4)
C2—C3—C4—O2	62.1 (4)	C4—N4—C13—C14	−145.7 (3)
O4—C3—C4—N4	5.3 (3)	C18—C13—C14—C15	1.1 (5)
C2—C3—C4—N4	−115.3 (3)	N4—C13—C14—C15	−178.7 (3)
N2—N1—C5—N3	−179.7 (3)	C13—C14—C15—C16	−1.5 (5)
N2—N1—C5—S1	0.4 (4)	C14—C15—C16—C17	1.2 (5)
C1—N3—C5—N1	−176.0 (3)	C14—C15—C16—Cl1	−178.6 (2)
C1—N3—C5—S1	3.9 (5)	C15—C16—C17—C18	−0.5 (5)
C6—S1—C5—N1	−0.4 (3)	Cl1—C16—C17—C18	179.4 (3)
C6—S1—C5—N3	179.6 (3)	C14—C13—C18—C17	−0.2 (5)
N1—N2—C6—C7	179.1 (3)	N4—C13—C18—C17	179.5 (3)
N1—N2—C6—S1	−0.2 (4)	C16—C17—C18—C13	−0.1 (5)
C5—S1—C6—N2	0.4 (3)		

### Hydrogen-bond geometry (Å, °)

**Table:**

*D*—H···*A*	*D*—H	H···*A*	*D*···*A*	*D*—H···*A*
O3—H3···N1^i^	0.82	1.95	2.733 (3)	159
N3—H3B···O3^ii^	0.86	2.35	3.061 (4)	140
O4—H4···O1^iii^	0.82	2.02	2.790 (3)	155
N4—H4A···O2^iv^	0.86	2.17	2.951 (4)	151

Symmetry codes: (i) −*x*+3/2, *y*−1/2, −*z*+1; (ii) −*x*+3/2, *y*+1/2, −*z*+1; (iii) −*x*+3/2, *y*+1/2, −*z*+2; (iv) *x*, *y*+1, *z*.
